# Supplementary material for: Development of deep learning-based detecting systems for pathologic myopia using retinal fundus images
Source: Commun Biol. 2021 Oct 26;4:1225. doi: 10.1038/s42003-021-02758-y (PMC8548495; doi:10.1038/s42003-021-02758-y)
Supplement: Supplementary file 2 — Supplementary Information [file 42003_2021_2758_MOESM2_ESM.pdf]

Supplementary Information

1. Supplementary Table 1. Summary of the training, validation, test and external validation dataset
2. Supplementary Table 2. Classification results for external validation dataset
3. Supplementary Table 3. Comparison of the performance of the different CNN-architectures in test dataset
4. Supplementary Figure 1. Precision recall (PR) curves of the DLSs in both test and external validation datasets
5. Supplementary Figure 2. The confusion matrixes of the DLSs in both test and external validation datasets
6. Supplementary Figure 3. The t-SNE plots of three DLSs in the test dataset.
7. Supplementary Figure 4. The example images of C0 to C4 MM and typical images of C3 and C4 MM in the test and external validation dataset
8. Supplementary Figure 5.The confusion matrices of other three CNN architectures evaluated in the test dataset
9. Supplementary Figure 6. The basic convolutional neural network (CNN) architecture and workflow of our DLSs

Supplementary Table 1.Summary of the training, validation, test and external validation dataset

	Training dataset (%)	Validation dataset (%)	Test dataset (%)	External validation dataset (%)
DLS for NPM/PM				
none PM	10237(89.0%)	2924(89.0%)	1462(89.0%)	800(85.4%)
Pathologic myopia	1265(11.0%)	360(11.0%)	180(11.0%)	137(14.6%)
DLS for ungradable/NPM/PM				
Ungradable images	631(5.2%)	181（5.2%）	90(5.2%)	63(6.3%)
Pathologic myopia(PM)	1263(10.4%)	361(10.4%)	181(10.4%)	137(13.7%)
none PM(NPM)	10236(84.4%)	2925(84.4%)	1462(84.4%)	800(80.0%)
DLS for Categories				
Category 0	466(18.9%)	139(19.6%)	88(23.6%)	35(14.4%)
Category 1	1107(45.1%)	316(44.7%)	158(42.5%)	121(49.6%)
Category 2	336(13.7%)	96(13.7%)	48(12.9%)	32(13.1%)
Category 3	316(12.9%)	90(12.7%)	45(12.1%)	33(13.5%)
Category 4	232(9.4%)	66(9.3%)	33(8.9%)	23(9.4%)

Abbreviations: DLS: deep learning system; PM: pathologic myopia; NPM: none pathologic myopia

Supplementary Table 2. Classification results for external validation dataset

Task of NPM/PM	AUC(95% CI)	Accuracy(95% CI)	Specificity(95% CI)	Sensitivity(95% CI)
DLS	0.989 (0.983 to 0.994)	96.3%(95.1 to 97.5)	90.5% (88.6 to 92.4)	97.3% (96.2 to 98.3)

Task of multiclass tasks	Macro-AUC	Accuracy(95% CI)	Quadratic-Weighted Kappa(95% CI)
DLS for ungradable/NPM/PM	0.967	97.8%(97.1 to 98.5)	0.905(0.863 to 0.948)
DLS for 5 myopic maculopathy categories	0.952	90.6%(89.2 to 92.0)	0.946(0.935 to 0.957)

Abbreviations: DLS: deep learning system; PM: pathologic myopia; NPM: none pathologic myopia

Supplementary Table 3. Comparison of the performance among the different CNN-architectures in test dataset

CNN-architecture for PM/NPM	AUC(95% CI)	Accuracy(95% CI)	Specificity(95% CI)	Sensitivity(95% CI)
Xception	0.993 (0.989 to 0.997)	97.7%(97.0 to 98.4)	97.2% (96.2 to 98.0)	97.7% (97.0 to 98.5)
ResNet50	0.991 (0.987 to 0.995)	97.3%(96.5 to 98.1)	95.6%(94.6 to 96.6)	97.5%(96.8 to 98.3)
DenseNet201	0.989 (0.984 to 0.994)	96.6%(95.7 to 97.5)	91.7%(90.3 to 93.0)	97.2%(96.4 to 98.0)
VGG16	0.989 (0.984 to 0.994)	96.5%(95.6 to 97.4)	92.8%(91.5 to 94.0)	96.9%(96.1 to 97.8)

CNN-architecture for ungradable/NPM/PM	Macro-AUC	Accuracy(95% CI)	Quadratic-Weighted Kappa(95% CI)
Xception	0.979	96.3%(95.1 to 97.5)	0.787(0.737 to 0.837)
ResNet50	0.963	95.7%(94.7 to 96.7)	0.771(0.702 to 0.822)
DenseNet201	0.941	94.4%(93.2 to 95.5)	0.733(0.681 to 0.786)
VGG16	0.928	94.2%(93.0 to 95.3)	0.713(0.659 to 0.767)

CNN-architecture for 5 MM categories	Macro-AUC	Accuracy(95% CI)	Quadratic-Weighted Kappa(95% CI)
Xception	0.978	97.6%(96.8 to 98.3)	0.990(0.985 to 0.994)
ResNet50	0.967	93.0%(91.8 to 94.2)	0.939(0.927 to 0.951)
DenseNet201	0.947	89.5%(88.0 to 91.0)	0.916(0.903 to 0.929)
VGG16	0.953	91.9%(90.6 to 93.2)	0.925(0.912 to 0.938)

Abbreviations: PM: pathologic myopia; NPM: none pathologic myopia; MM: myopic maculopathy

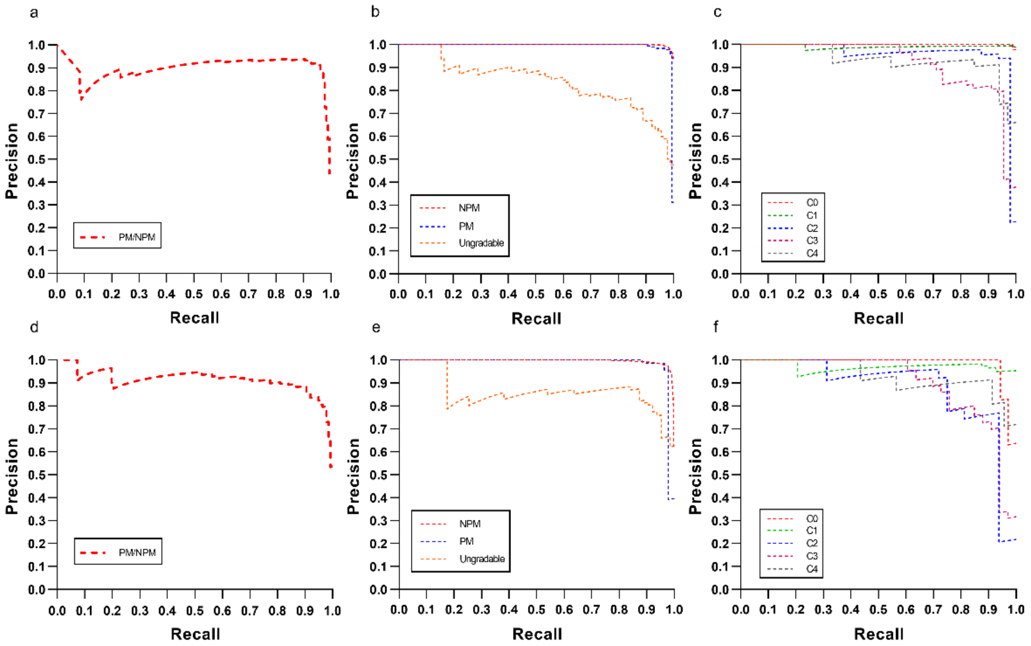

Supplementary Figure 1. Precision recall (PR) curves of the DLSs in both test and external validation datasets. (a) The PR curve of the DLS for NPM/PM in the test dataset. (b) The PR curve of the DLS for three-class task in the test dataset. (c) The PR curve of the DLS for five-class task in the test dataset. (d) The PR curve of the DLS for NPM/PM in the external validation dataset. (e) The PR curve of the DLS for three-class task in the external validation dataset. (f) The PR curve of the DLS for five-class task in the external validation dataset. NPM: none pathologic myopia. PM: pathologic myopia. C: Category.

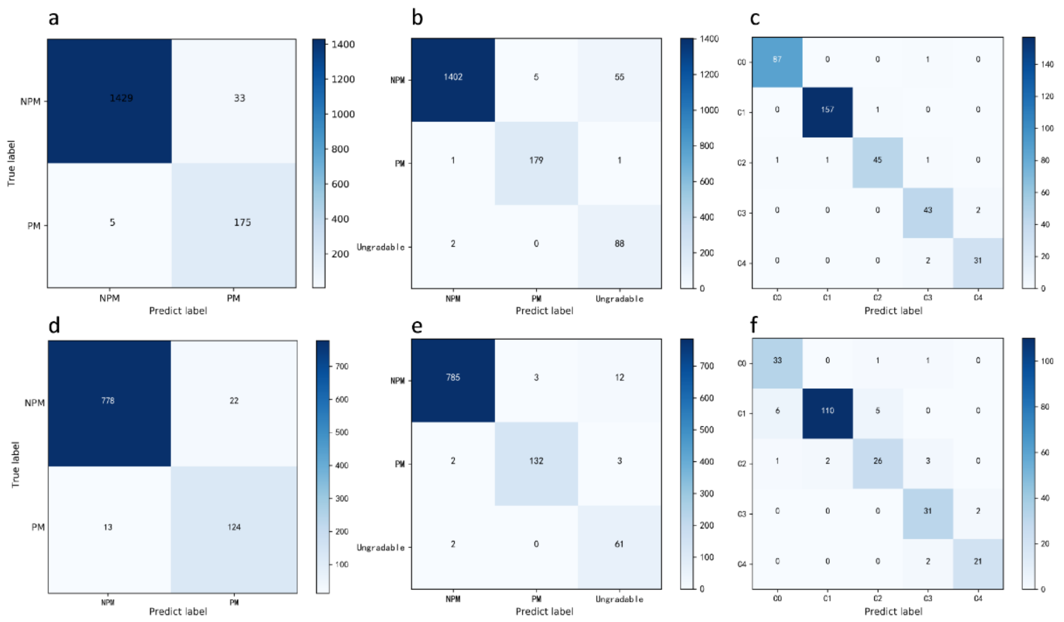

Supplementary Figure 2. The confusion matrixes of the DLSs in both test and external validation datasets. (a) The confusion matrix of the DLS for NPM/PM in the test dataset. (b) The confusion matrix of the DLS for three-class task in the test dataset. (c) The confusion matrix of the DLS for five-class task in the test dataset. (d) The confusion matrix of the DLS for NPM/PM in the external validation dataset. (e) The confusion matrix of the DLS for three-class task in the external validation dataset. (f) The confusion matrix of the DLS for five-class task in the external validation dataset. NPM: none pathologic myopia. PM: pathologic myopia. C: Category.

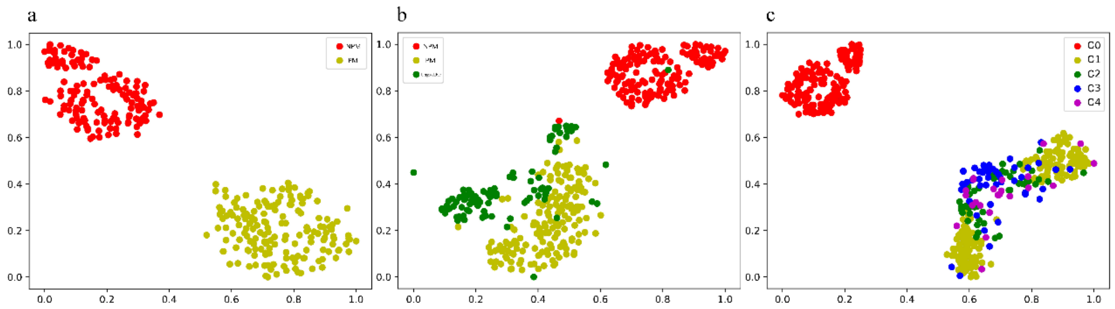

Supplementary Figure 3. The t-SNE plots of three DLSs in the test dataset. (a) The t-SNE plot for NPM/PM. (b) The t-SNE plot for three-class task. (c) The t-SNE plot for five-class task.

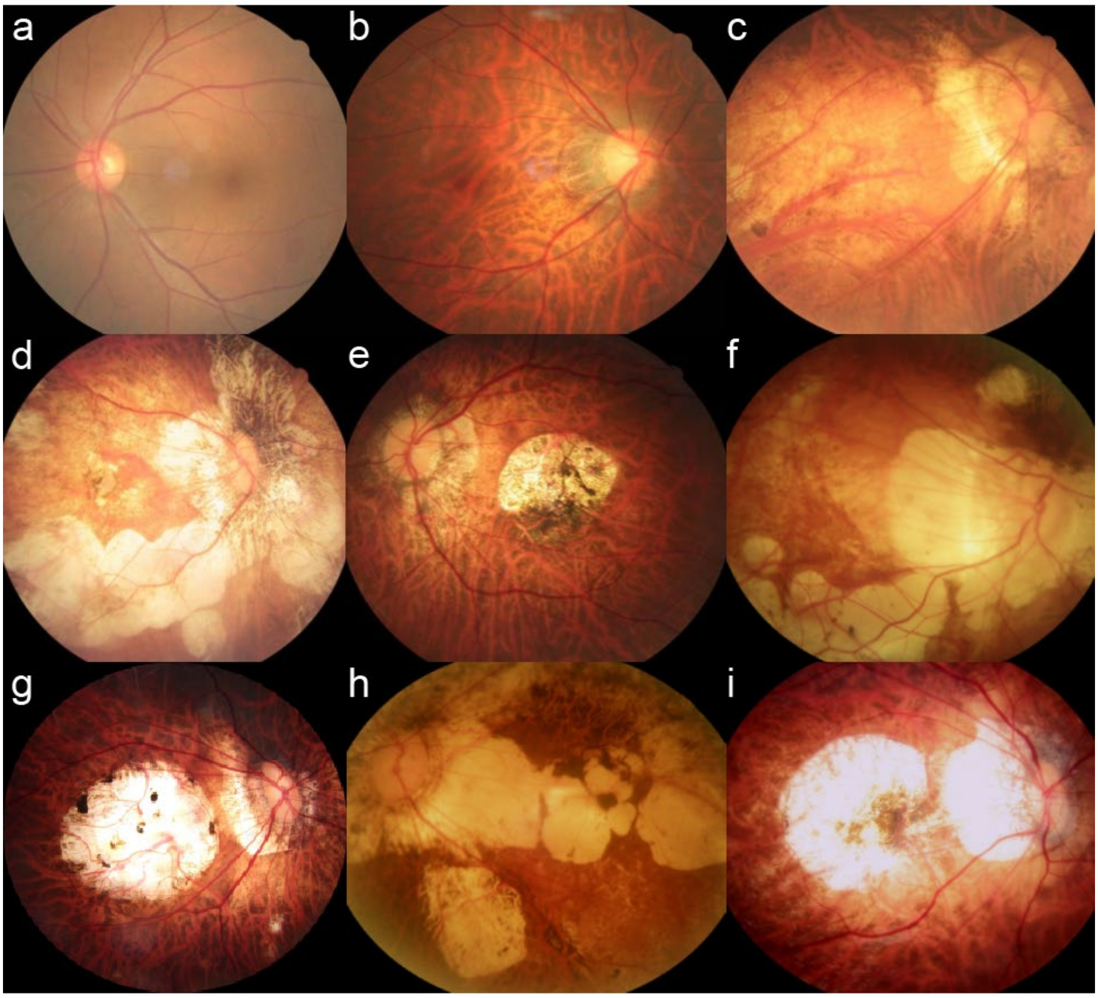

Supplementary Figure 4. The example images of C0 to C4 MM and typical images of C3 and C4 MM in the test and external validation dataset. (a) The example images of C0. (b) The example image of C1 (tessellated fundus). (c) The example image of C2 (diffuse chorioretinal atrophy). (d) The example image of C3 (patchy chorioretinal atrophy). (e) The example image of C4 (macular atrophy). (f) The typical image of C3 in the test dataset. (g) The typical image of C4 in the test dataset. (h) The typical image of C3 in the external validation dataset. (i) The typical image of C4 in the external validation dataset.

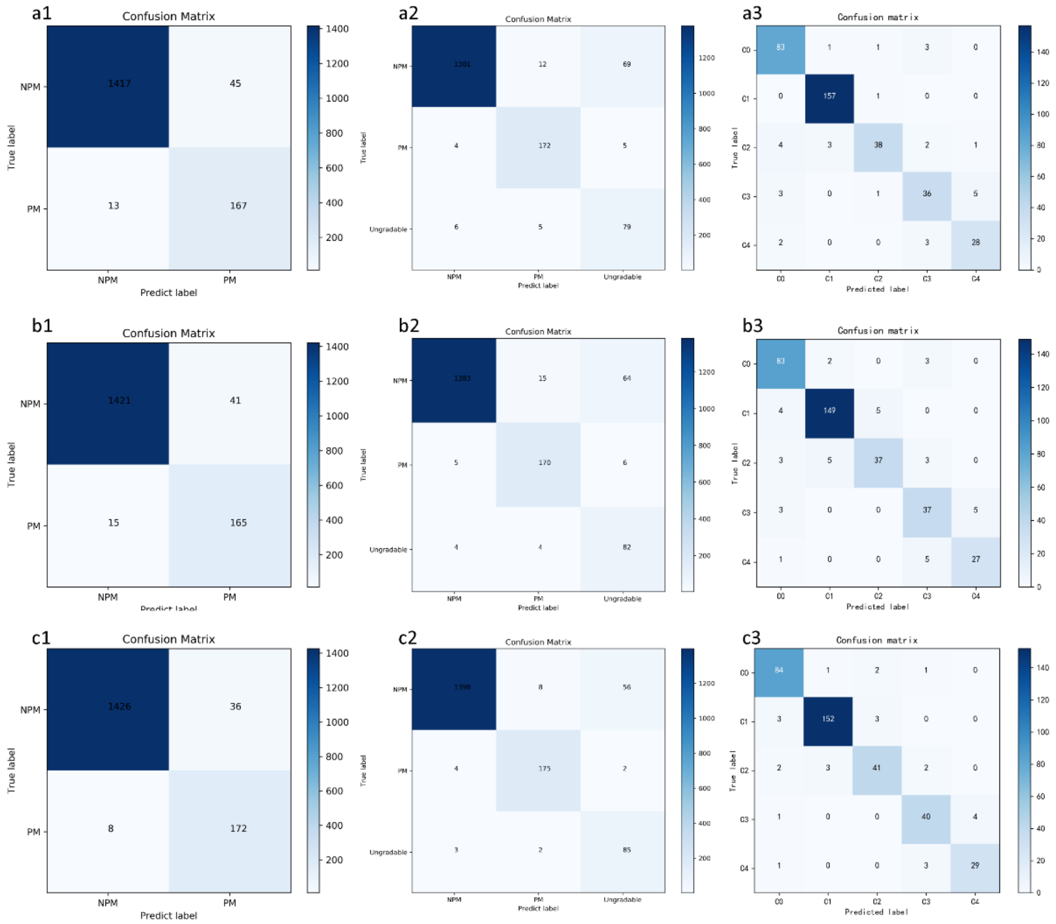

Supplementary Figure 5. The confusion matrices of other three CNN architectures evaluated in the test dataset. (a1-a3) The confusion matrices of VGG16. (b1-b3) The confusion matrices of DenseNet201. (c1-c3) The confusion matrices of ResNet50. NPM: none pathologic myopia. PM: pathologic myopia. C: Category.

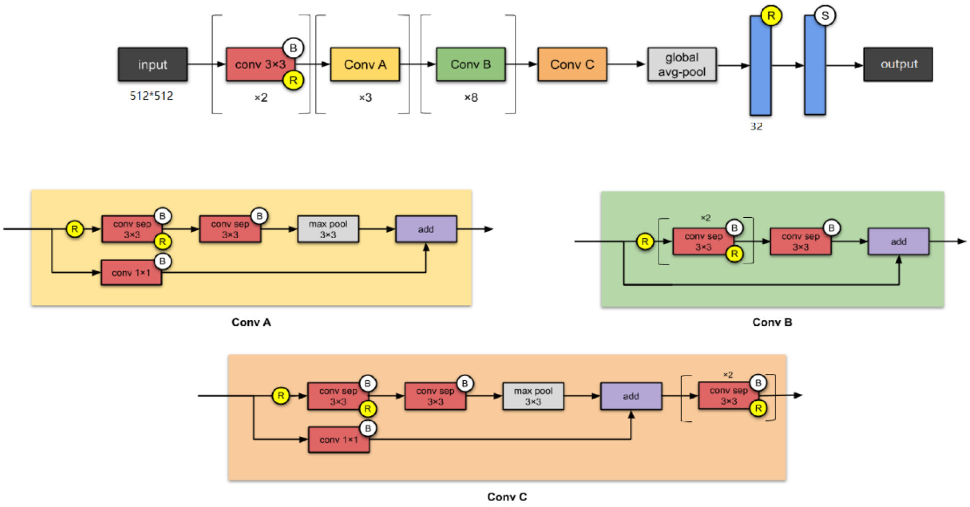

Supplementary Figure 6. The basic convolutional neural network (CNN) architecture and workflow of our DLSs.

Conv: Convolution layers. conv sep: Separable Convolution layers. R: Rectified linear unit (ReLU). B: Batch normalization. S: Softmax.
