# Supplementary material for: Development of deep learning-based detecting systems for pathologic myopia using retinal fundus images
Source: Commun Biol. 2021 Oct 26;4:1225. doi: 10.1038/s42003-021-02758-y (PMC8548495; doi:10.1038/s42003-021-02758-y)
Supplement: Supplementary file 3 — Description of Additional Supplementary Files [file 42003_2021_2758_MOESM3_ESM.pdf]

Description of Additional Supplementary Files

File name: Supplementary Data 1

Description: Source data underlying Figures 2 and Supplementary Figure 1.
