# Supplementary material for: Development of deep learning-based detecting systems for pathologic myopia using retinal fundus images
Source: Commun Biol. 2021 Oct 26;4:1225. doi: 10.1038/s42003-021-02758-y (PMC8548495; doi:10.1038/s42003-021-02758-y)
Supplement: Supplementary file 5 — Reporting Summary [file 42003_2021_2758_MOESM5_ESM.pdf]

nature portfolio

Corresponding author(s): Wei Han

Last updated by author(s): Sep 22, 2021

Reporting Summary

Nature Portfolio wishes to improve the reproducibility of the work that we publish. This form provides structure for consistency and transparency in reporting. For further information on Nature Portfolio policies, see our Editorial Policies and the Editorial Policy Checklist.

Statistics

For all statistical analyses, confirm that the following items are present in the figure legend, table legend, main text, or Methods section.

n/a	Confirmed	
☐	☒	The exact sample size (*n*) for each experimental group/condition, given as a discrete number and unit of measurement
☐	☒	A statement on whether measurements were taken from distinct samples or whether the same sample was measured repeatedly
☐	☒	The statistical test(s) used AND whether they are one- or two-sided<br>*Only common tests should be described solely by name; describe more complex techniques in the Methods section.*
☒	☐	A description of all covariates tested
☒	☐	A description of any assumptions or corrections, such as tests of normality and adjustment for multiple comparisons
☐	☒	A full description of the statistical parameters including central tendency (e.g. means) or other basic estimates (e.g. regression coefficient) AND variation (e.g. standard deviation) or associated estimates of uncertainty (e.g. confidence intervals)
☐	☒	For null hypothesis testing, the test statistic (e.g. *F*, *t*, *r*) with confidence intervals, effect sizes, degrees of freedom and *P* value noted<br>*Give P values as exact values whenever suitable.*
☒	☐	For Bayesian analysis, information on the choice of priors and Markov chain Monte Carlo settings
☒	☐	For hierarchical and complex designs, identification of the appropriate level for tests and full reporting of outcomes
☒	☐	Estimates of effect sizes (e.g. Cohen's *d*, Pearson's *r*), indicating how they were calculated

Our web collection on statistics for biologists contains articles on many of the points above.

Software and code

Policy information about availability of computer code

Data collection	No software was used.
Data analysis	According to the reference standard in binary task, the performance of the selected general ophthalmologist, retinal specialist, and the DLSs in the test and external validation dataset was calculated using the indices of sensitivity, specificity, accuracy and area under the receiver operating curve (AUC). For the multi-class tasks, the area under the macro average of ROC (macro-AUC) for each class in one-vs-all manner, quadratic-weighted kappa score and accuracy were calculated. We also demonstrated the confusion matrices for the multi-class classification tasks. Additionally, the Clopper-Pearson method was used to calculate the 95% CI. Statistical data were analyzed using Sigma Plot 14.0 and Python 3.7.3.

For manuscripts utilizing custom algorithms or software that are central to the research but not yet described in published literature, software must be made available to editors and reviewers. We strongly encourage code deposition in a community repository (e.g. GitHub). See the Nature Portfolio guidelines for submitting code & software for further information.

Data

Policy information about availability of data

All manuscripts must include a data availability statement. This statement should provide the following information, where applicable:

- Accession codes, unique identifiers, or web links for publicly available datasets
- A description of any restrictions on data availability
- For clinical datasets or third party data, please ensure that the statement adheres to our policy

The datasets generated and/or analyzed during the current study are available from the corresponding author on a reasonable request. Correspondence and requests for data materials should be addressed to Wei Han (hanweidr@zju.edu.cn). All datasets are stored at our FTP server

Field-specific reporting

Please select the one below that is the best fit for your research. If you are not sure, read the appropriate sections before making your selection.

- [x] Life sciences
- [ ] Behavioural & social sciences
- [ ] Ecological, evolutionary & environmental sciences

For a reference copy of the document with all sections, see nature.com/documents/nr-reporting-summary-flat.pdf

Life sciences study design

All studies must disclose on these points even when the disclosure is negative.

Sample size	A total dataset was generated, containing 17,330 color retinal fundus images from 13869 myopia patients aged 14–75 years obtained from the eye center of the First Affiliated Hospital of School of Medicine, Zhejiang University between July 2016 and June 2018. The desktop nonmydriatic retinal cameras and digital retinography systems (Canon) were used to capture the retinal fundus images, which were maculalutea-centered 45° color fundus photographs. The pupil dilation was decided by the examiners depending on the patient's ocular condition
Data exclusions	The binary task: Images obtained from eyes with previous history of refractive surgery and the ungradable images were excluded.<br><br>The three-class task: All the images involved in the binary task and the ungradable images excluded in the binary task were all included in the three-class task.<br><br>The five-class task: Images obtained from eyes with previous history of refractive surgery and the ungradable images were excluded
Replication	In the present study, three DLSs were trained using a novel convolutional neural network (CNN) architecture, Xception. In this novel architecture, Inception modules were replaced with depthwise separable convolutions to handle spatial-correlations and cross-channel correlations independently. It is believed that an independent processing of corss-channel correlations would better address the tasks involved in this study. The DLSs were trained to handle binary or multiclass classification tasks: (i) a binary system of none pathologic myopia/ pathologic myopia (NPM/PM), (ii) a three-class system of ungradable images/NPM/PM, and (iii) five-class system of five MM categories. We used a two-step training process. In the first training step, only the last full connection layer was fine-tuned and the weights of the basic Xception were not updated. In the second training step, the whole model was fine-tuned and the weights of all the layers were updated. We also used the early stopping strategy. Training was stopped if the validation loss did not decrease in 5 consecutive epochs during the training process. We also recruited 1,000 images from 738 patients aged 17–79 years (Table 1) in another hospital in Shanghai to verify the reproducibility.
Randomization	To develop the three DLSs, images were randomly assigned to training (70%), validation (20%), and test (10%) datasets. Each image was always taken from a different eye and was only allowed to exist in one dataset, so that no same image would appear in both the training and test datasets.
Blinding	Before the DLSs showed their performances in test datasets, graders were masked to the results of DLSs.

Reporting for specific materials, systems and methods

We require information from authors about some types of materials, experimental systems and methods used in many studies. Here, indicate whether each material, system or method listed is relevant to your study. If you are not sure if a list item applies to your research, read the appropriate section before selecting a response.

Materials & experimental systems

n/a	Involved in the study
x	Antibodies
x	Eukaryotic cell lines
x	Palaeontology and archaeology
x	Animals and other organisms
	x Human research participants
x	Clinical data
x	Dual use research of concern

Methods

n/a	Involved in the study
x	ChIP-seq
x	Flow cytometry
x	MRI-based neuroimaging

Human research participants

Policy information about studies involving human research participants

Population characteristics	A total dataset, containing 17,330 color retinal fundus images from 13869 myopia patients aged 14–75 years (mean age 49.5 years, 66.1% female), was generated
Recruitment	Color retinal fundus images were obtained from the eye center of the First Affiliated Hospital of School of Medicine, Zhejiang University between July 2016 and June 2018
Ethics oversight	In this study, the use of retinal fundus images was approved by the Ethics Committee of First Affiliated Hospital, School of Medicine, Zhejiang University (Hangzhou, Zhejiang, China) and adhered to the tenets of the Declaration of Helsinki .Approval ID: NO. 2020-693.

Note that full information on the approval of the study protocol must also be provided in the manuscript.
